# Daily transcriptomes of the copepod *Calanus finmarchicus* during the summer solstice at high Arctic latitudes

**DOI:** 10.1038/s41597-020-00751-4

**Published:** 2020-11-24

**Authors:** Laura Payton, Céline Noirot, Claire Hoede, Lukas Hüppe, Kim Last, David Wilcockson, Elizaveta A. Ershova, Sophie Valière, Bettina Meyer

**Affiliations:** 1 Institute for Chemistry and Biology of the Marine Environment, Carl von Ossietzky University of Oldenburg, Oldenburg, 26111 Germany; 2 Section Polar Biological Oceanography, Alfred Wegener Institute Helmholtz Centre for Polar and Marine Research, Bremerhaven, 27570 Germany; 3 Plateforme bio-informatique GenoToul, MIAT, INRAE, UR875 Mathématiques et Informatique Appliquées Toulouse, F-31326 Castanet-Tolosan, France; 4 Helmholtz Institute for Functional Marine Biodiversity (HIFMB) at the University of Oldenburg, Oldenburg, 26111 Germany; 5 Scottish Association for Marine Science, Oban, Argyll PA37 1QA UK; 6 Institute of Biological, Environmental, and Rural Sciences, Aberystwyth University, Aberystwyth, SY23 3DA UK; 7 Department for Arctic and Marine Biology, Faculty for Biosciences, Fisheries and Economics, UiT The Arctic University of Norway, Tromsø, N-9037 Norway; 8 Shirshov Institute of Oceanology, Russian Academy of Sciences, 36 Nakhimova Avenue, Moscow, Russian Federation 117997 Russia; 9 Plateforme Génomique, INRAE US 1426 GeT-PlaGe, Centre INRAE de Toulouse Occitanie, 24 Chemin de Borde Rouge, Auzeville, 31326 Castanet-Tolosan cedex France

**Keywords:** Transcriptomics, Ecophysiology, Molecular ecology

## Abstract

The zooplankter *Calanus finmarchicus* is a member of the so-called “Calanus Complex”, a group of copepods that constitutes a key element of the Arctic polar marine ecosystem, providing a crucial link between primary production and higher trophic levels. Climate change induces the shift of *C. finmarchicus* to higher latitudes with currently unknown impacts on its endogenous timing. Here we generated a daily transcriptome of *C. finmarchicus* at two high Arctic stations, during the more extreme time of Midnight Sun, the summer solstice. While the southern station (74.5 °N) was sea ice-free, the northern one (82.5 °N) was sea ice-covered. The mRNAs of the 42 samples have been sequenced with an average of 126 ± 5 million reads (mean ± SE) per sample, and aligned to the reference transcriptome. We detail the quality assessment of the datasets and the complete annotation procedure, providing the possibility to investigate daily gene expression of this ecologically important species at high Arctic latitudes, and to compare gene expression according to latitude and sea ice-coverage.

## Abstract (summary)

**Table:** 

Measurement(s)	transcriptome • sequence feature annotation
Technology Type(s)	RNA sequencing • sequence annotation
Factor Type(s)	location • time
Sample Characteristic - Organism	Calanus finmarchicus
Sample Characteristic - Environment	polar
Sample Characteristic - Location	Arctic Ocean

Machine-accessible metadata file describing the reported data: 10.6084/m9.figshare.13008035

## Background & Summary

The copepod *Calanus finmarchicus* (Crustacea, Copepoda) is a key zooplankton species in the northern Atlantic food web as it converts sugars from algae into energy rich lipids that sustain higher consumers including marine fish larvae and seabirds^1–3^. Its high abundance and biomass also makes it an important contributor to ocean carbon flux^4^. The species inhabits a large latitudinal range from ~40° up to 80° N^5^. However, recent findings show that *C. finmarchicus* is undergoing temperature driven geographical shifts northwards because of climate change^6–8^, the effects of which are at their most extreme in the Northern Atlantic and Barents Sea. Therefore, the copepods will experience a change between the photoperiods they are adapted to at lower latitudes and the extreme high-latitude photoperiods. Photoperiodic variation is particularly pronounced in the Arctic with rapid change over short latitudinal ranges. The impact of such extreme photoperiods on non-endemic species is unknown, and the northward expansion of organisms at high latitudes may be limited by the adaptive capacity of their endogenous timing systems to extreme photoperiods^8,9^.

Endogenous timing systems, or biological clocks, are ubiquitous ancient and highly adaptive mechanisms enabling organisms to track and anticipate environmental cycles and prepare biological processes accordingly^10,11^. Since the identification of circadian clock genes in *C. finmarchicus*^12^, studies have shown that this species possesses a functional clock that might be involved in the timing of both diel and seasonal events, such as the ecologically and biogeochemically important diel vertical migration (DVM)^13^ or diapause^14^. However, the Arctic environment is characterized by dramatic seasonality resulting in permanent illumination during Midnight Sun and permanent darkness during Polar Night^15^. As the circadian clock is entrained and synchronized by daily light/dark cycles, the persistence of daily biological processes in Arctic organisms during the absence of those remains uncertain^16,17^, as well as the consequences for newcomer species due to global warming^8,9^. Moreover, the Arctic is characterized by strong fluctuations in sea ice-cover, reflecting on biotic and abiotic factors, such as species communities and interactions or light penetration^18,19^.

Copepods are among the important non-model invertebrates for which genomic resources are still limited, one barrier being that many species, including *C. finmarchicus*, have large genomes^20,21^. The *de novo* transcriptome of *C. finmarchicus*^22^ represents a useful resource for assessing the impact of global warming in this species of high ecological interest. In addition to differential gene expression analyzes, RNA sequencing has increased the ability to study the expression of rhythmically expressed mRNAs^23–25^. Indeed, at the molecular level, the endogenous clock machinery drives the rhythmic expression of downstream genes whose rhythmic translation and function ultimately underlie daily oscillations at cellular and organismal levels^25^. Note that in the field, environmental cycles also directly generate rhythms independently from the clock. Thus, temporal transcriptomic studies allow a major breakthrough in the understanding of daily dynamics of biological processes in the field.

In this study, we performed RNA sequencing on temporally collected *in situ* samples to generate a daily transcriptome of *C. finmarchicus* in the high Arctic during summer solstice period when the sun remains high above the horizon with minimal altitude variation. Sampling of *C. finmarchicus* stage V copepodites was performed at 4 h intervals within a 24 h cycle at two ocean stations along a latitudinal gradient. The northern station (82.5 °N, Nansen Basin) was characterized by sea ice-coverage, while the southern one (74.5 °N, Barents Sea) was sea ice-free. In addition to providing the raw data, we describe its quality assessment and the alignment to the reference transcriptome to verify reliability and determine transcript quantification. Finally a complete annotation is performed and two normalized datasets are provided for further transcriptomic data exploration of this species.

## Methods

### Sampling design

**Fig. 1:**
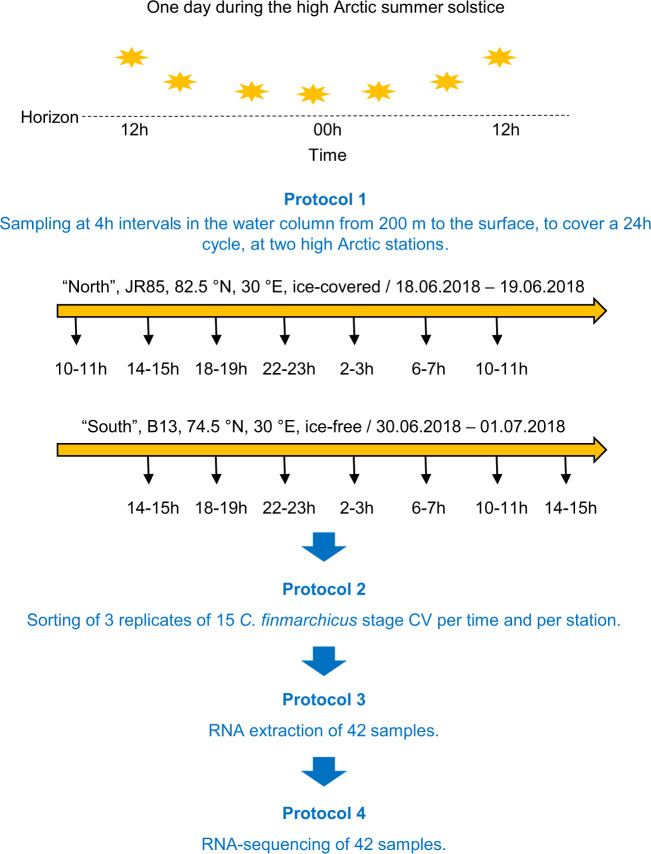
Overview of the experimental workflow used to generate the transcriptomic data output (all times noted in local time (UTC + 2)). Sample details are available in Supplementary Table 1.

The sampling strategy was specifically designed for the detection of rhythmic transcripts^25,26^ although it does not exclude classic differential expression analysis^27^. Sampling design and analysis strategy are presented in Fig. 1, Table 1 and Supplementary Table 1. Sampling covered a complete 24 h cycle at 4 h intervals, resulting in seven time points per station. At each station, samplings were performed at similar time intervals of: 14–15 h, 18–19 h, 22–23 h, 2–3 h, 6–7 h, and 10–11 h (all times noted in local time (UTC + 2)). Sampling at “North” station, JR85, started on 18th June (3 days before the summer solstice) at 10–11 h and ended on 19th June at 10–11 h. Sampling at “South” station, B13, started on 30th June (9 days after the summer solstice) at 14–15 h and ended on 1st July at 14–15 h. At each timepoint the water column was sampled from 200 m to the surface with vertical hauls of a WP2 plankton net (opening ∅: 57 cm, net length: 236 cm, mesh size: 200 µm) with a meshed bucket cod end (mesh size: 200 µm) at a speed of 0.5 m*s^−1^. Transferring the animals from the net into the stabilization solution was done within less than 12 minutes for all samplings. A 12 h period of incubation at 2–4 °C was allowed to soak the samples thoroughly with the RNAl*ater* stabilization solution (Ambion, UK) before they were transferred to −80 °C for further transport and storage.

**Table 1:** Summary of sampling and sequencing strategy. Details are available in Supplementary Table 1.

Station	Number of timepoint	Sampling frequency	Total duration of sampling	Number of replicate per timepoint	Total number of samples	Sequencing strategy	Reads	Platform
“North”	7	4 h	24 h	3	21	RNA-seq	paired-end 2 × 150 pb	Illumina NovaSeq
“South”	7	4 h	24 h	3	21	RNA-seq	paired-end 2 × 150 pb	Illumina NovaSeq

### Sites description

Sampling has been conducted during Cruise JR17006 of the *RRS James Clark Ross* in summer 2018 at two stations along a latitudinal gradient. The station “North” was sea ice-covered and located in the Nansen Basin (JR85; 82.56°N, 30.85°E). The station “South” was sea ice-free and located in the southern Barents Sea (B13; 74.5°N, 30°E). Water depth at “North” was 3700 m and at “South” was 360 m. The sun’s altitude was always above the horizon but still showed diel oscillations of altitude above the horizon from 16 ° at midnight to 30.9 ° at midday at “North”, and from 7.7 ° at midnight to 38.6 ° at midday at “South” at the times of sampling (all times noted in local time (UTC + 2)). Sites were exposed to semidiurnal tide regimes, i.e., 2 tides per day, with a maximum amplitude of ± 0.47 m at JR85 and ± 0.36 m at B13 at times of sampling. Maps with the location of the sea ice edge at the time of sampling at “North” are available from the *meereisportal*^28^ (https://data.meereisportal.de/gallery/index_new.php?active-tab1=method&ice-type=satellite&satellite=A&region=n&resolution=daily&minYear=2018&minMonth=6&minDay=18&maxYear=2018&maxMonth=6&maxDay=19&showMaps=y&dateRepeat=n&submit2=display&lang=en_US&active-tab2=satellite). Modeled data of sun altitude were obtained from the United States Naval Observatory (https://aa.usno.navy.mil/data/docs/AltAz.php, USNO, USA). Information on the tidal dynamics have been drawn from the TPX08 model^29^ by using the OTPS package (Tidal Prediction Software, http://www-po.coas.oregonstate.edu/~poa/www-po/research/po/research/tide/index.html), via the mbotps program^30^ (MB-System). Solar altitude, tidal height and sea-ice cover during the sampling campaign at both latitudes are detailed in Supplementary Table 2. Temperature, pressure (depth), conductivity (salinity), oxygen saturation (SBE 43, Sea-Bird Electronics) and Chlorophyll *a* (Chl *a*) fluorescence (Aquatracka III fluorometer, Chelsea Technologies Group, UK) were measured from the surface to 200 m depth and are available in Hueppe *et al*.^31^.

### Copepod sorting

Copepods were sorted at 2 °C under a stereo microscope for species (*C. finmarchicus*) and stage (*CV*). To distinguish *C. finmarchicus* from its closely related congener *C. glacialis*, morphological indicators were used, in particular the redness of the antenna, which has been shown to be a good indicator in the regions of sampling^32^; see also the molecular validation of morphological identification, below. For each timepoint and station, 3 replicates of 15 *C. finmachicus* CV were sorted. The choice to pool 15 individuals was made to (1) get the sufficient amount of RNA required for RNA sequencing and quantitative real-time PCR analyses and (2) increase the number of individuals (315 copepods per station in total) thereby decreasing the effect of individual variability.

### RNA extraction

Each replicate was distributed to a 2 ml Precellys® homogenization tube (Bertin Instruments, France), containing a mix of 1.4 mm and 2.8 mm ceramic beads and homogenized in 600 µl of TRIzol® reagent (ThermoFisher Scientific, USA) with a Precellys® 24 Tissue Homogenizer (Bertin Instruments, France), using two times 15 sec. of homogenization at 5000 rpm with a 10 sec. break between. For RNA extraction, a Phenol/Chloroform based single-step extraction in combination with a spin column based solid phase extraction (Direct-zol™ RNA MiniPrep Kit, Zymo Research, USA) was used. Genomic DNA was removed by DNase I digestion on column as part of the RNA extraction kit and total RNA was eluted in ultra-pure water. A portion of the RNA of each of the samples was used to investigate relative expression of 8 candidate genes with SYBRGreen based quantitative real-time PCR (qPCR) on candidate genes, using the 2^−∆Ct^ method^32^ and the geometric mean of *elongation factor 1α* and *16 s rRNA* as reference, as described by Hueppe *et al*.^31^. Another portion of each samples was send to GeT-PlaGe core facility in dried-ice for RNA sequencing.

### RNA sequencing

RNA sequencing was performed at the GeT-PlaGe core facility, INRAE Toulouse. The 42 RNA sequencing libraries were prepared according to Illumina’s protocols using the Illumina TruSeq Stranded mRNA sample prep kit to analyse mRNA. Briefly, mRNA were selected using poly-T beads. Then, RNA were fragmented to generate double stranded cDNA and adaptors were ligated to be sequenced. 11 cycles of PCR were applied to amplify libraries. Library quality was assessed using a Fragment Analyser (Advanced Analytical Technologies, Inc., Iowa, USA) and libraries were quantified by qPCR using the Kapa Library Quantification Kit (Roche). RNA sequencing experiments have been performed on a NovaSeq S4 lane (Illumina, California, USA) using a paired-end read length of 2 × 150 pb with the Illumina NovaSeq Reagent Kits.

### Reads alignment and quantification

42 RNA sequencing libraries were obtained (Fig. 1, Table 1, and Supplementary Table 1). The number of paired reads per library was between 74 million and 276 million with an average of 126 ± 5 million (mean ± SE) reads. The RNA sequencing libraries reads quality were evaluated using FastQC^33^. Contamination was checked by aligning reads against *E. coli*, Yeast and PhiX genomes.

The *Calanus finmarchicus de novo* transcriptome^22^, based on different life stages and deposited to Bioproject PRJNA236528, was used as the reference transcriptome. It is composed of 206,012 contigs and presents good results of quality assessment, with a nearly complete BUSCO set^22,34^. Reads were aligned to the *de novo* transcriptome with BWA-MEM (http://bio-bwa.sourceforge.net/bwa.shtml). Quantification was performed with SAMtools^35^ idxStats to generate the quantification matrix. The matrix was filtered with edgeR^36^ and only contigs with more than 1 CPM (Count Per Million) in at least one sample were kept, providing a matrix of 76,550 contigs. Information on the datasets resulting from this study is available in Table 2.

**Table 2:** List of available datasets related to the study (NCBI Bioproject PRJNA628886^40^ and figshare collection 5127704^41^).

DOI	Availability	File Name	Description	File format
—	https://www.ncbi.nlm.nih.gov/bioproject/PRJNA628886	SRR11748365.fastq.gz to SRR11748406.fastq.gz	Raw RNA sequencing data	fastq
10.6084/m9.figshare.c.5127704	10.6084/m9.figshare.c.5127704	PRJNA628886_raw_quantification_206K.tsv.gz	Raw count matrix of the 206,012 contigs	tsv
		76k_ids_list.txt	List of identifiers corresponding to the 76,550 contigs after filtering	txt
		PRJNA628886_quantification_downsampled_76k.tsv.gz	Down-sampling normalized quantification matrix	tsv
		PRJNA628886_quantification_RLE_76k.tsv.gz	RLE normalized quantification matrix	tsv
		Interprot_annot_206K.gff3	Gff3 InterProScan annotation of the 206,012 contigs	gff3
		Interprot_annot_206K.tsv	Tabulated InterProScan annotation of the 206,012 contigs	tsv
		diamond_annotation_206k.tsv	Tabulated DIAMOND annotation matrix of the 206,012 contigs (NR,swissprot,trembl)	tsv
		diamond_annotation_76k.tsv	Tabulated DIAMOND annotation matrix of the 76,550 contigs (NR,swissprot,trembl)	tsv

Details are available in Supplementary Table 1.

### Annotation

We provided different annotations for all further analysis. Contigs were aligned with DIAMOND^37^ on NR (2019-09-29), Swissprot and Trembl (2018-12) to retrieve corresponding best annotations. An annotation matrix was then generated by selecting the best hit for each database if: i) the percent of the query length covered by the alignment was higher than 60%; ii) the percent of the subject length covered by the alignment was higher than 40%; iii) the percent of identity of the alignment was higher than 40%. Contigs were also processed with InterProScan^38^ to scan InterProScan signatures. A GO was assigned to each contig with an InterProScan hit containing a GO annotation. Information on the datasets resulting from this study is available in Table 2. Note that a previous annotation of *Calanus finmarchicus* reference transcriptome^22^ against Non-redundant (NR) protein database is also available at 10.5061/dryad.11978.

### Normalization

Two normalizations are proposed (down-sampling normalization and RLE normalization) but the choice of normalization depends on the analysis required downstream. For a rhythmic analysis, we suggest down-sampling the mapped reads to the lowest number among the 42 samples (down-sampling normalization), i.e. to 70.4 million properly mapped reads per sample for all samples (after filtering), in order to adjust for differences in sequencing depth among samples^23,25,39^. This was performed with StreamSampler.jar (https://github.com/shenkers/sampling). EdgeR^36^ was used to perform RLE normalization, since it is more appropriate for differential expression analysis. Information on the datasets resulting from this study is available in Table 2.

## Data Records

Raw reads were gathered in the NCBI BioProject PRJNA628886^40^ which includes all BioSamples used for the study (Table 2, Supplementary table 1). We also provide the following in figshare collection 5127704^41^ (Table 2): the quantification matrix for the 206,012 contigs; the list of identifiers corresponding to the 76,550 contigs after filtering; the two suggested normalization matrices (down-sampling and RLE) and; the datasets annotations (DIAMOND annotation matrix, InterProScan annotation, GO association).

## Technical Validation

### Molecular validation of morphological identification

Since *C. finmarchicus’* Arctic congener *C. glacialis* also occurs in the region of sampling and differences between the species can be very subtle^42^, morphological identification was validated by molecular species identification on a subset of samples from the same stations^21,43^. DNA was extracted from individual copepods using the HotShot method^44^, and the species-specific nuclear insertion/deletion (InDel) marker G-150 was amplified using a modified protocol from Smolina *et al*.^45^. Identification was done by accessing the size of the resulting amplicon via electrophoresis on a 2% agarose gel. Results have shown that 99% of the individuals identified as *C. finmarchicus* by the morphological identification method were also clearly identified as *C. finmarchicus* by the molecular identification method, while 0.1% were not clearly identified and 0.7% were identified as the Arctic congener *Calanus glacialis* (n = 305 individuals).

### Extraction and RNA integrity

RNA extraction procedures were performed with randomization of samples to ensure reliable and unbiased data production. RNA purity was assessed by OD measurements with a NanoDrop 8000 spectrophotometer (ThermoFisher Scientific, USA), and all 260/280 and 260/230 OD ratio was superior to 1.9. RNA integrity was evaluated with a Fragment Analyzer (Advanced Analytical Technologies, Inc., Iowa, USA; RNA Kit (15nt) Standard Sensitivity, Agilent). Due to a non-conventional 28 S/18 S ribosomal ratio in this species, sample quality was evaluated on the electropherogram^46^. No degradation in the inter region was observed. Total RNA samples were stored at −80 °C.

### Raw reads assessment and quantification overview

**Fig. 2:**
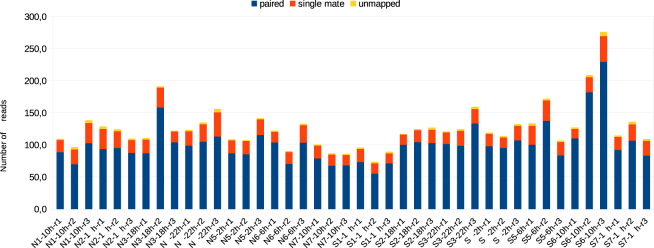
Mapping statistics of the 42 samples against the set of 206,012 contigs. Number of million (M) reads: paired, single mate, and unmapped. Details are available in Supplementary Table 3.

All samples passed the FastQC^33^ “base quality control”. No relevant contamination hit was found after the alignment against *E. coli*, Yeast and PhiX. The mapping rate against the reference transcriptome^22^ of 206,012 contigs was higher than 72.4% for properly paired reads and higher than 93.6% considering both paired and single mate reads, validating the raw reads quality (Fig. 2, Supplementary Table 3). Furthermore, over the 42 samples, the maximal percentage of multi-mapped alignment is of 3.31% (Fig. 2, Supplementary Table 3).

**Fig. 3:**
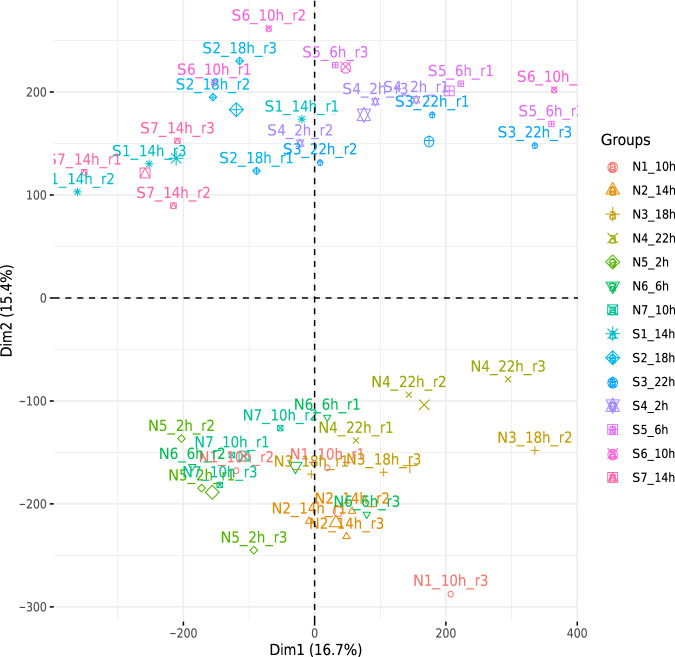
Principal component analysis of the 42 samples based on expression levels of the whole transcriptome (206,012 contigs). “Groups” grouping the 3 replicates per time and per station.

For an overview of the quantification matrix, a principal component analysis (PCA) was generated on the raw pseudo-count (log2 (count + 1)) non-normalized matrix (Fig. 3). Results showed a clear separation between samples from “North” and “South” stations, indicating environmental variations that might be due to latitude and/or sea ice-coverage.

### Filtering

Of the 206,012 transcripts, 37% (76,550) were expressed above the threshold of 1 CPM. This result corroborates previously observed results on the *C. finmarchicus* transcriptome^22^. Thus a large proportion of the whole contigs (63%) exhibited an extremely low level of expression, representing only 1.32 ± 0.04% of total aligned reads at “North”, and 1.27 ± 0.05% at “South” (Table 3, Supplementary Table 4).

**Table 3:** Average number (million, mean ± SE) of alignments per station and percentage of alignments discarded by filtering contigs with very low expression.

Station	Average number of aligned reads on full dataset (206,012 contigs)	Average number of aligned reads after filtering (76,550 contigs)	Percentage of aligned reads discarded by filtering
“North”	117.9 ± 5.4	116.3 ± 5.3	1.32 ± 0.04%
“South”	129.9 ± 9.3	128.3 ± 9.2	1.27 ± 0.05%

Details are available in Supplementary Table 4.

### Contigs annotation

By selecting the best hit for each database, the annotation matrix generated with Diamond^37^ has led to 36,274 and 22,527 contigs with an annotation in at least one database out of the 206,012 and 76,550 contigs respectively (Table 4). Moreover, the number of unique hits for each database is always lower than the number of contigs annotated by the respective database, highlighting the contigs’ functional redundancies.

**Table 4:** Number of contigs annotated with DIAMOND against NR, TREMBL and Swissprot and number of unique hits in the target database.

Dataset	Database	Number of contigs	Number of unique hit in target database
206,012 contigs	NR	32,637	16,165
	Trembl	28,845	15,518
	Swissprot	11,668	5,486
	With an annotation in at least one database	36,274	—
76,550 contigs	NR	21,171	11,045
	Trembl	17,988	10,051
	Swissprot	8,456	4,343
	With an annotation in at least one database	22,527	—

The InterProScan annotation provided annotations from many protein signature databases. The main results are presented in Table 5 and Supplementary Table 5. A GO was attributed to 65,924 contigs over the whole transcriptome (206,012 contigs), while 33,057 contigs out of the 76,550 contigs with an expression level higher than 1 CPM in at least one sample had a GO annotation (Table 5, Supplementary Table 5).

**Table 5:** Number of contigs with an InterProScan annotation and details on main features.

Dataset	Main programs			Extracted GO
	PFAM	TMHMM	getorf	
206,012 contigs	63,661	123,542	192,688	65,924
76,550 contigs	34,558	48,682	73,272	33,057

Details are available in Supplementary Table 5.

### Quantitative real-time PCR data for normalization verification

The relative expression of six core circadian clock genes (*clock*, *cycle*, *period1*, *timeless*, *cryptochrome2*, *vrille*) and 2 circadian clock-related genes (*cryptochrome1* and *doubletime2*) was investigated by quantitative real-time PCR and are available in Supplementary Table 6, allowing the verification of RNA sequencing normalization for further investigations. Regarding the two normalizations, the down-sampling normalization was selected for a rhythmic analysis based on concordant temporal expression profiles with qPCR data (using RAIN algorithm^47^), while the RLE normalization has been validated for differential expression analysis of the mean level of expression between stations, using the 21 samples of each stations as replicates.

## Usage Notes

We present here the first *in situ* daily transcriptomes from the high Arctic, where molecular investigations of biological rhythms are exceptionally limited^15,16^. The samplings have been realized during drastic Polar photic conditions, i.e. the summer solstice, when daily oscillations of the Sun are minimal, high in the sky and always above the horizon^15^. The proposed datasets are thus novel and of interest due to the unique geographical location and time of year, the ecological importance of *C. finmarchicus*, and the rigorous temporal sampling strategy. Another strength of this dataset is the high depth of the RNA sequencing, with an average of 126 ± 5 million of reads (mean ± SE) per sample, which optimizes the detection of rhythmic transcripts^25^ in a species with a large genome^20,21^. Finally, the elaborate annotation of the large transcriptome is now publicly available and is thus accessible for further research.

The sampling strategy is optimized for rhythmic analysis, and particularly adapted for RAIN algorithm analysis^23,25,47^. Moreover, dataset allows powerful differential gene expression analysis using the 21 samples per station as replicates providing time-integrated detection of differentially expressed genes in *C. finmarchicus* with latitude/sea ice-cover. With climate driven environmental changes, this dataset ultimately constitutes new insights into transcriptomic regulation in the northward migrating copepod *C. finmarchicus*.

## Supplementary information

Supplementary tables

## Data Availability

Parameters to software tools involved are described in the following paragraph. FastQC: version 0,11,2, --nogroup --casava. DIAMOND: version v0.9.22, parameters: -f 6 qseqid qlen qcovhsp pident score evalue length sseqid slen stitle. InterProScan: version 5.29–68.0, --goterms -t n -dp -f TSV, gff3 parameters. BWA: version 0.7.17, standard parameters, mem algorithm. SAMtools programs (view, sort, index and idxStats, flagstat): version 1.8, standard parameters. EdgeR: version 3.26.5. StreamSampler.jar: version 1.0.

## References

[1] Prokopchuk I, Sentyabov E (2006). Diets of herring, mackerel, and blue whiting in the Norwegian Sea in relation to *Calanus finmarchicus* distribution and temperature conditions. ICES J. Mar. Sci..

[2] Beaugrand G, Brander KM, Alistair Lindley J, Souissi S, Reid PC (2003). Plankton effect on cod recruitment in the North Sea. Nature.

[3] Berge J, Gabrielsen TM, Moline M, Renaud PE (2012). Evolution of the Arctic *Calanus* complex: an Arctic marine avocado?. J. Plankton Res..

[4] Archibald KM, Siegel DA, Doney SC (2019). Modeling the Impact of Zooplankton Diel Vertical Migration on the Carbon Export Flux of the Biological Pump. Glob. Biogeochem. Cycles.

[5] Helaouët P, Beaugrand G (2007). Macroecology of *Calanus finmarchicus* and *C. helgolandicus* in the North Atlantic Ocean and adjacent seas. Mar. Ecol. Prog. Ser..

[6] Murphy EJ (2016). Understanding the structure and functioning of polar pelagic ecosystems to predict the impacts of change. Proc. R. Soc. B Biol. Sci..

[7] Reygondeau G, Beaugrand G (2011). Future climate-driven shifts in distribution of *Calanus finmarchicus*. Glob. Change Biol..

[8] Saikkonen K (2012). Climate change-driven species’ range shifts filtered by photoperiodism. Nat. Clim. Change.

[9] Huffeldt NP (2020). Photic Barriers to Poleward Range-shifts. Trends Ecol. Evol..

[10] Emerson KJ, Bradshaw WE, Holzapfel CM (2008). Concordance of the Circadian Clock with the Environment Is Necessary to Maximize Fitness in Natural Populations. Evolution.

[11] Bradshaw WE, Holzapfel CM (2010). What Season Is It Anyway? Circadian Tracking vs. Photoperiodic Anticipation in Insects. J. Biol. Rhythms.

[12] Christie AE, Fontanilla TM, Nesbit KT, Lenz PH (2013). Prediction of the protein components of a putative *Calanus finmarchicus* (Crustacea, Copepoda) circadian signaling system using a de novo assembled transcriptome. Comp. Biochem. Physiol. Part D Genomics Proteomics.

[13] Häfker NS (2017). Circadian Clock Involvement in Zooplankton Diel Vertical Migration. Curr. Biol. CB.

[14] Häfker NS (2018). *Calanus finmarchicus* seasonal cycle and diapause in relation to gene expression, physiology, and endogenous clocks. Limnol. Oceanogr..

[15] Schmal, C., Herzel, H. & Myung, J. Clocks in the Wild: Entrainment to Natural Light. *Front. Physiol*. **11** (2020). 10.3389/fphys.2020.00272 PMC7142224 32300307

[16] Abhilash L, Shindey R, Sharma VK (2017). To be or not to be rhythmic? A review of studies on organisms inhabiting constant environments. Biol. Rhythm Res..

[17] Bertolini E (2019). Life at high latitudes does not require circadian behavioral rhythmicity under constant darkness. Curr. Biol..

[18] David C, Lange B, Rabe B, Flores H (2015). Community structure of under-ice fauna in the Eurasian central Arctic Ocean in relation to environmental properties of sea-ice habitats. Mar. Ecol. Prog. Ser..

[19] Falk-Petersen S (1998). Lipids and fatty acids in ice algae and phytoplankton from the Marginal Ice Zone in the Barents Sea. Polar Biol..

[20] Bron JE (2011). Observing copepods through a genomic lens. Front. Zool..

[21] Choquet Marvin *et al*. Towards population genomics in non-model species with large genomes: a case study of the marine zooplankton *Calanus finmarchicus*. *R. Soc. Open Sci.* **6**, 180608 (2019). 10.1098/rsos.180608 PMC6408391 30891252

[22] Lenz PH (2014). De Novo Assembly of a Transcriptome for *Calanus finmarchicus* (Crustacea, Copepoda) – The Dominant Zooplankter of the North Atlantic Ocean. PLOS ONE.

[23] Hughes ME (2017). Guidelines for Genome-Scale Analysis of Biological Rhythms. J. Biol. Rhythms.

[24] Mermet J, Yeung J, Naef F (2017). Systems Chronobiology: Global Analysis of Gene Regulation in a 24-Hour Periodic World. Cold Spring Harb. Perspect. Biol..

[25] Li J, Grant GR, Hogenesch JB, Hughes ME (2015). Considerations for RNA-seq analysis of circadian rhythms. Methods Enzymol..

[26] Ness-Cohn E, Iwanaszko M, Kath W, Allada R, Braun R (2020). TimeTrial: An Interactive Application for Optimizing the Design and Analysis of Transcriptomic Times-Series Data in Circadian Biology Research. J. Biol. Rhythms.

[27] Payton L (2017). Remodeling of the cycling transcriptome of the oyster *Crassostrea gigas* by the harmful algae Alexandrium minutum. Sci. Rep..

[28] Grosfeld, K. *et al*. Online Sea-Ice Knowledge and Data Platform. https://www.meereisportal.de/ (2016).

[29] Egbert GD, Erofeeva SY (2002). Efficient Inverse Modeling of Barotropic Ocean Tides. J. Atmospheric Ocean. Technol..

[30] Caress, D. W. & Chayes, D., N. MB-System Version 5.5.2284. *Open source software distributed from the MBARI and L-DEO web sites*. (2016).

[31] Hüppe L (2020). Evidence for oscillating circadian clock genes in the copepod *Calanus finmarchicus* during the summer solstice in the high Arctic. Biol. Lett..

[32] Livak KJ, Schmittgen TD (2001). Analysis of relative gene expression data using real-time quantitative PCR and the 2^−ΔΔCT^ method. Methods.

[33] Andrews S (2010). FastQC: a quality control tool for high throughput sequence data..

[34] Tarrant AM, Nilsson B, Hansen BW (2019). Molecular physiology of copepods - from biomarkers to transcriptomes and back again. Comp. Biochem. Physiol. Part D Genomics Proteomics.

[35] Li H (2009). The Sequence Alignment/Map format and SAMtools. Bioinformatics.

[36] Robinson MD, McCarthy DJ, Smyth G (2010). K. edgeR: a Bioconductor package for differential expression analysis of digital gene expression data. Bioinformatics.

[37] Buchfink B, Xie C, Huson DH (2015). Fast and sensitive protein alignment using DIAMOND. Nat. Methods.

[38] Jones P (2014). InterProScan 5: genome-scale protein function classification. Bioinformatics.

[39] Koike N (2012). Transcriptional Architecture and Chromatin Landscape of the Core Circadian Clock in Mammals. Science.

[40] (2020). NCBI Sequence Read Archive.

[41] Payton L (2020). figshare.

[42] Nielsen TG, Kjellerup S, Smolina I, Hoarau G, Lindeque P (2014). Live discrimination of *Calanus glacialis* and *C. finmarchicus* females: can we trust phenological differences?. Mar. Biol..

[43] Choquet M (2017). Genetics redraws pelagic biogeography of *Calanus*. Biol. Lett..

[44] Truett GE (2000). Preparation of PCR-quality mouse genomic DNA with hot sodium hydroxide and tris (HotSHOT). BioTechniques.

[45] Smolina I (2014). Genome- and transcriptome-assisted development of nuclear insertion/deletion markers for *Calanus* species (Copepoda: Calanoida) identification. Mol. Ecol. Resour..

[46] DeLeo, D. M., Pérez-Moreno, J. L., Vázquez-Miranda, H. & Bracken-Grissom, H. D. RNA profile diversity across arthropoda: guidelines, methodological artifacts, and expected outcomes. *Biol. Methods Protoc*. **3** (2018). 10.1093/biomethods/bpy012 PMC6994094 32161805

[47] Thaben PF, Westermark PO (2014). Detecting rhythms in time series with RAIN. J. Biol. Rhythms.

